# Coping with Fear of and Exposure to Terrorism among Expatriates

**DOI:** 10.3390/ijerph14070808

**Published:** 2017-07-19

**Authors:** Nicholas J. Beutell, Marianne M. O’Hare, Joy A. Schneer, Jeffrey W. Alstete

**Affiliations:** 1 School of Business, Iona College, New Rochelle, NY 10801, USA; jalstete@iona.edu; 2 College of Education and Human Services, Seton Hall University, South Orange, NJ 07079, USA; mohare2345@gmail.com; 3 College of Business Administration, Rider University, Lawrenceville, NJ 08648, USA; schneer@rider.edu

**Keywords:** expatriates, international assignees, stress, fear, terrorism, hostile environments coping, repatriates, duty of care, virtual reality, post-traumatic stress disorder

## Abstract

This paper examines existing research on the impact of terrorism on expatriate coping strategies. We consider pre-assignment fear of terrorism, in-country coping strategies, and anxiety and posttraumatic stress disorder (PTSD) associated with repatriation. The extant research is small but growing. Our model for expatriate coping at the pre-departure, in-country, and repatriation stages includes strategies specific to each stage. Preparation using proactive coping, systematic desensitization, problem and emotion focused coping, social support, and virtual reality explorations are recommended. Selecting expatriate candidates who are well-adjusted, emotionally intelligent, and possessing good coping skills is essential for successful assignments in terror-prone regions.

## 1. Introduction

As company needs for successful expatriates are increasing, so is the proliferation of terrorist activities and fear of terrorist threats in many parts of the world. Fear has already been identified as a problem for expatriate workers [1], including a specific fear of terrorism or terrorist threats [2]. To complicate these matters, terrorism can be extremely disruptive to international business activities [3]. We know that the threat of terrorism increases stress [4] as well as employee anger, frustration, and negative emotions [5]. Since many of the extant models of expatriation are based on stress theory [6], our primary interest is in coping with the stress of different stages of expatriation. Few studies have focused specifically on expatriate coping with the fear of terrorism and specific strategies for dealing with terroristic threats and attacks. We examine the anticipation of, and possible posting to, a country where there is a threat of terrorist activity. As Bader, Reade and Froese [6] have argued, expatriate stressors in terror-endangered countries go beyond regular expatriate cultural adjustment processes. The threat of terrorism is qualitatively different from the typical stressors affecting expatriate adjustment. This paper considers the psychological aspects of coping with the fear of and exposure to terrorism. Very little research has focused on individuals and terrorism in a business context [7] using psychological stress and coping models. Our approach is intended to complement the growing research on expatriates in hostile environments [7,8,9]. Specifically, we propose: (1) to examine the literature on expatriate coping in relation to terrorism; (2) present a heuristic model of coping strategies for pre-assignment, on-ground, and repatriation stages; and (3) suggest directions for future research on coping with terrorist threats and behaviors.

## 2. What Is Terrorism?

While an exact definition of terrorism has been elusive there are a number of agreed upon aspects that comprise terroristic behavior. As Miranda [10] has stated “the 21st century is seeing an explosion of asymmetrical and unconventional warfare of which terrorism is an ascending form” (p. 49). Essentially, terrorism is psychological warfare [11]. We focus on international terrorism, although, in the case of expatriates, terrorism could be domestic, international, or both. International terrorism means activities with the following three characteristics:

◾ Involve violent acts or acts dangerous to human life;
◾ Appear to be intended to intimidate or coerce a civilian population; to influence the policy of a government by intimidation or coercion; or to affect the conduct of a government by mass destruction, assassination, or kidnapping; and
◾ Transcend national boundaries in terms of the means by which they are accomplished, the persons they appear intended to intimidate or coerce, or the locale in which their perpetrators operate or seek asylum.

Furthermore, most of the terrorism today appears to be based on the concept of a just war theory and the Islamic norms of jihad and shahadat to justify terrorism [12], although opinions vary on the divergence between jihad and terrorism in today’s context [13]. It should also be noted that terrorism and the threat of terrorism are less predictable and more frequent as time goes on since individuals and small groups have the capability of initiating attacks [14]. A topical study suggests that “global terrorism has increased in recent years and occurs in unexpected places, at unexpected times, with unexpected casualties” [15] (p. 3). In effect, the threat of terrorism is omnipresent.

## 3. What Is an Expatriate?

The answer to this seemingly innocuous question is more complicated than it would appear. Recent work by McNulty and Brewster [16], however, suggests the following definition:

legally working individuals who reside temporarily in a country of which they are not a citizen in order to accomplish a career-related goal, being relocated abroad either by an organization, by self-initiation or directly employed within the host-country (p. 20).

This definition focuses on business expatriates and is consistent with the field of international human resource management. It excludes other groups such as tourists or immigrants. Workers assigned to foreign countries often experience a stress-induced reaction, culture shock, as they are confronted with the realities of their new environment [17]. However, successful expatriates offer many potential benefits, such as the “transfer of managerial and technological knowledge, better control of foreign subsidiaries, improved communication, and more secure business transactions” (p. 135) [18]. Therefore, it is important that businesses stay informed about the rapidly developing and changing nature of international work assignments, currently pressing stressors such as terrorism, and methods to effectively prepare expatriates to manage these potential problems.

We recognize that there are other types of international assignees in addition to expatriates. These include corporate executives who engage in short but frequent global travel. Such temporary travel is different from spending prolonged periods in a location where one feels the threat of terrorism. Our definition of expatriates requires an extended duration foreign assignment (typically from six months to five years). This allows us to conceptualize coping strategies that may be effective before the foreign assignment, during the assignment, and after completion of the assignment in a region perceived as terror-prone.

## 4. Coping with Exposure to Terrorism: Models and Research

With the globalization of markets and the rise in terrorism, living and working in a foreign country can be dangerous and stressful for expatriates and other international assignees. As a result, researchers have been giving attention to the behaviors that enable expatriates to live and work in countries that are under constant threat of terrorism [19]. In addition, attention is being given to the traits and coping strategies of expatriates. It has been suggested that organizations should select expatriates on such personality traits as emotional stability, being outgoing and agreeable, and being open to new experiences [19]. However, it is also important to understand the coping strategies used by expatriates; that prepare and allow them to live and work in a foreign country and, also deal with the challenges of living in an environment in which there is constant imminent danger [20,21].

Coping is defined as “constantly changing cognitive and behavioral efforts to manage specific external and internal demands that are appraised as taxing or exceeding the resources of the person” [22] (p. 141). This is consistent with other research on coping among expatriates [23]. We examine the extant literature focusing on expatriates along with some of the models (e.g., conservation of resources) that support previous empirical work focusing specifically on expatriates as well as other work that is germane to expatriates. Lazarus and Folkman [22] identify two broad types of coping responses: emotion-focused and problem-focused. Emotion-focused coping tries to reduce negative emotional reactions to events (like terrorism) that are not controlled by the individual. Problem-focused coping is an individual’s attempt to remove or reduce the source of stress. This is a more proactive attempt to deal with the stressor(s). This distinction between emotion-focused and problem-focused coping has been used in previous work examining expatriate coping [23,24]. Some research suggests that emotion-focused coping is more effective in dealing with terror events since such occurrences are unpredictable, uncontrollable, and produce chronic stress [25]. It has also been suggested that coping with uncontrollable events such as terrorism, may require a blending of problem-focused and emotion-focused approaches [25].

The growing body of research that focuses on coping strategies and behaviors of expatriates frequently uses the Lazarus and Folkman [22] model of stress, appraisal, and coping. This model views coping as a transactional process: that is, once an encounter is appraised by the expatriate as a threat, the expatriate utilizes cognitive and/or behavioral strategies intended to manage, alter, or regulate distress. Coping is an ongoing process whereby expatriates use cognitive or behavioral factors to exert control over a threatening situation [11]. A reaction to a threat can, in fact, become a stressor itself.

Stahl and Caligiuri [23], for example, investigated the coping behaviors of expatriate managers, using the Lazarus and Folkman [22] framework, to understand the manager’s cross-cultural adjustment. The results were mixed. There was a relatively high level of adjustment, using problem-solving coping strategies [22], but contingent on position level and country of assignment [23]. They did not focus on the effects of coping with the constant threat of terrorism but their results indicate differences in coping strategies based on country and level in the organization.

Giorgi, Montani, Fiz-Perez, Arcangeli and Mucci [21] did explore fear of terrorism with respect to mental health and adjustment to the foreign country. During periods of stress, an expatriate suffers health problems and, also, anxiety related to expatriation itself [21]. The risk factors associated with health problems and anxiety include: being involved in accidents, poor living conditions, unsafe working conditions, disease contagion, fear of kidnapping, violence, and terrorist attacks. Giorgi, Montani, Fiz-Perez, Arcangeli and Mucci [21] built on the Lazarus and Folkman [22] model of stress and coping and examined the emergence of fear of expatriation due to the risk factors in 265 Italian expatriates from one company. The authors note that fear of expatriation, in their study, was associated with mental health problems, such as stress, anxiety, loneliness, and homesickness. It was also noted that expatriates cannot count on family or trusted friends for support [21]. The findings were supportive of Lazarus and Folkman [22] and affective events theory [26]. Such results indicated that the fear of expatriation generalized to further fears in the workplace and make it unlikely that the expatriate will adjust to the destination or the work [21]. Although this study was concerned with the expatriate’s fear of terrorism, it did not address the issue of coping with the imminent threat of a terrorist attack while the expatriate is living in the foreign country.

An interesting and potentially useful framework that might be applied to the expatriate’s living and working abroad, is the idea of proactive coping [26]. Although their work is not directly focusing on expatriates, Aspinwall and Taylor [26] explain proactive coping as the efforts that a person would take in order to prepare for a perceived possible threat, before it occurs, in an effort to prevent or modify it, if it should it occur [26]. Hobfoll et al. [27] allude to a similar idea in their study of terrorism in Israel, with Jewish and Palestine citizens (non-expatriates). Aspinwall and Taylor [26] discussed the differences among proactive coping, “coping”, and “anticipatory coping” [28]. Proactive coping differs from “coping” (an attempt to master, tolerate, or reduce perceived, potential threats) [29] and “anticipatory coping” (preparation for dealing with the consequences of an upcoming, potential threat [28,29] in that the stressful event has not occurred. That is, according to Aspinwall and Taylor [26], proactive coping does not address a specific event. Therefore, it is important to develop skills that enable the person (expatriate) to identify potential sources of threat and prepare for their possibility or even inevitability. It is suggested that, even in the case of an unavoidable stressful event, proactive coping will be associated with more positive adjustment than will the person who did not engage in preparatory activities [26].

Hobfoll, Canetti-Nisim and Johnson [27] examined the factors that contribute to stress and resiliency in those who are chronically exposed to terrorist attacks. Their sample consisted of 905 Jewish and Palestinian adult citizens of Israel. They proposed a model depicting the impact of terrorism on the resource gains and losses of those who live in a country in which there is frequent exposure to terrorism and a constant threat. It was predicted and supported that, although more Jews have been injured or killed in Israel than Palestinians, Palestinians would feel the greater psychological distress from the impact of terrorism. Consistent with the conservation of resources (COR) [30] model, it was believed that the ethnic minority group (Palestinians) would have fewer resources (social support, psychological, and economic) needed to combat the impact of terrorism [28].

According to Hobfoll, Canetti-Nisim and Johnson [27], the COR [30] model can be used to predict outcomes after a terrorist attack, war, or disaster. It is an integrated model of stress that suggests that individuals seek to acquire and maintain resources that can be material, emotional, psychological, temporal or economic. Stress occurs when there is a loss of resources, or a threat of resource loss. They concluded that the resource losses of family, friends, economic viability, and social connections, of those who face on-going terrorist attack and threat, were critical in predicting post-traumatic stress disorder (PTSD) and psychological distress [28]. Based on Hobfoll, Canetti-Nisim and Johnson [27], it can be thought that those individuals who are well integrated in their communities and have strong, supportive social relationships might be more resilient. Social support might prevent negative psychological consequences, like PTSD. However, as Hobfoll, Canetti-Nisim and Johnson [27] note, the loss of these resources could result in greater psychological distress and PTSD. They add that “interventions can target resources in order to prevent their loss, or alter environmental contingencies” increasing resiliency. Furthermore, “public officials and media can present information about appropriate forms of social support and effective coping that aid resiliency” [27].

Similar to a proactive model of coping [26], the authors [28] recommend counseling that pertains to terrorism and focuses on self-efficacy [31], and that could protect against negative consequences after an attack or threat. Interestingly, the authors also noted that few of the Israelis in this study (less than 1%) experienced death or injury of members of their nuclear family. This fact might serve to distance the Jewish people, in the study, from the direct impact of terrorism. Creating distance could lessen the Jewish people’s concern or they might just take the constant terror activity for granted. That is, they may decide to accept their life as is [32]. Or, the Jewish people in their sample might choose to use a type of coping strategy defined by Hall [33] as “Type II” coping, personal role redefinition, in which a person redefines his/her expectation of the self and his/her own behavior in a given situation. For example, the person in the chronically terrorist threatened situation might cope by thinking that s/he does not have to act or behave differently because s/he has been safe so far.

In addition, while living in constant threat of attack and exposure to terrorism could challenge a sense of self-efficacy, and increase the fear of loss of loved ones or economic resources, it might, on the other hand, enhance one’s sense of self-efficacy or, at least, create a buffer to the stress consequences, or, perhaps, increase an inoculation effect or self-control [34]. The act of terrorism might be seen as personal (perpetrator is more important than those who are harmed) and the consequences would be harmful (loss of life, resources, and dehumanization) and the person might feel emotionally numb. However, if the terrorism is not experienced as personal (no one in the immediate family was injured or killed), the resulting outcome may be that the person can cope and overcome adversity [34]. Living with or surviving a terrorist attack, like war or trauma, can be transformational [34]; the survivor experience in a war-torn or terrorist-threatened country might result in positive aspects of stress, as well as negative ones.

Much more research is needed to understand the benefit of social relationships, the role of self-efficacy, and resilience in those who are in the wake of terror. In addition, the Hobfoll, Canetti-Nisim and Johnson [27] study investigated the impact of terrorism on those who are citizens of an area that is under constant threat of attack. Note that the participants were not expatriates. More research is needed on the impact of terrorism and the coping behaviors/strategies of those who are temporarily living and working in a foreign country and who are or might be risking their lives.

## 5. Coping during Different Stages of Expatriation

**Figure 1:**
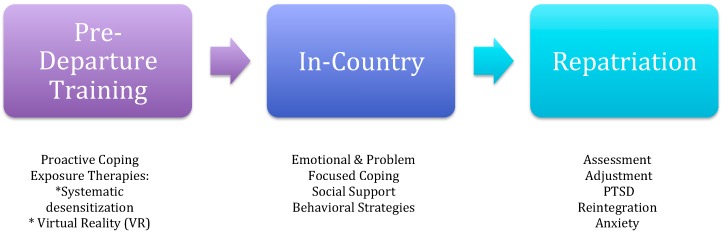
Model of expatriate coping in terror-endangered countries.

Based on the foregoing, we have developed a model showing coping strategies at different stages in the expatriation process: pre-departure; in-country experiences; and, repatriation (Figure 1). Our stage view is important since coping is a dynamic process that develops over time [24]. The stages in the model are conceptually distinct yet not independent: coping resources and strategies affect subsequent stages even though different strategies may be needed. Coping with the fear of terrorism requires different coping mechanisms than those required during a terror attack. Coping with repatriation after living in a terror-endangered environment could take months, or even years, as symptoms of anxiety disorders and PTSD may not be manifested immediately on returning to one’s home country. This implies that effective coping at one stage can increase coping success at the next stage even though different strategies are utilized. We envision the model as a temporal scheme with transitions between each stage. There is a psychological difference as well as a locational change between stages. Thus, the transition between each stage will pose different types of threats (and opportunities) and coping skills. There is some evidence to suggest differential success of coping strategies based on the in-country and repatriation stages [35] of our model.

Prior to pre-departure training, the company must be clear on the qualities that they are seeking in candidates for assignment to terror prone regions. Some authors have discussed the notion that only single employees be considered for such postings [36]. This seems a bit antiquated but it does highlight the risks of deploying families in such environments. “Companies should conduct preventive screening to identify the human resources to be sent to foreign countries, favoring those who have demonstrated both a highly qualified professionalism in their field and robust mental health” [21] (p. 9). Mental health issues appear to heighten fears regarding terrorism in expatriation [17]. Qualifications and mental health considerations are appropriate, yet an individual’s sensitivity to terrorism means that companies must be careful not to oversell such assignments by using pressure, including possible bullying (e.g., [37]), that can increase employee fears, including fear of economic loss [38]. Mentally healthy individuals may decide that a high-risk deployment is not a good fit for them or their family.

Similar findings in the practitioner literature indicate that such assignments are not a good match for all who express interest. Although overall adjustment and mental health [20] should be considered, some candidates are at higher risk when assigned to terror-prone regions. Grove and Hallowell [39] offer the following: (1) our assumption is that your candidates will be technically competent; (2) our research reveals why certain people are especially at risk abroad; (3) our assessment methods identify high-risk candidates for expatriation; and (4) somehow, high-risk candidates must be eliminated from consideration. There is a need for open and frank discussions about candidate and family suitability for assignments with higher risk of terrorist events.

### 5.1. Pre-Departure Coping Strategies

In addition to “regular” pre-departure training efforts, we believe that expatriates who will be deployed in terror-prone regions should be taught proactive coping skills (Figure 1). Proactive coping occurs prior to “coping”. As Aspinwall and Taylor [26] point out, proactive coping “involves the accumulation of resources and the acquisition of skills that are not designed to address any particular stressor but to prepare in general” (p. 417). As noted previously, it is important to develop skills that enable the expatriate to identify potential sources of threat and prepare for their possibility. Even in the case of an unavoidable stressful event, a person who utilized proactive coping will experience more positive adjustment than will the person who did not engage in preparatory activities. For example, we know that bombs are the most frequent type of terrorist threat [40]. However, only 2% of terrorist bomb threats actually result in an attack [41]. Bomb threats must be taken seriously but there will be many false positives. This can help the expatriate to gain perspective and to feel more in control of their situation.

As Giorgi, Montani, Fiz-Perez, Arcangeli and Mucci [21] note:

First, we believe that the subjects in the process of leaving their own country should be mentally healthy and not feeling frightened by either the place of destination or the assigned tasks. According to Lazarus and Folkman (1984), if an expatriate is worried and anxious, it is less likely that he/she will ever adjust. Therefore, it is essential to help expatriates to prevent the development of any type of fear (p. 9).

Interestingly Giorgi, Montani, Fiz-Perez, Arcangeli and Mucci [21] pointed out, those who are considering employment in certain organizations, might, also, fear expatriation, which can generalize to fear in the workplace. As a result, it is unlikely that s/he will adjust to the destination or the work [21]. Furthermore, it added a new component, the generalized fear of expatriation, which also needs to be explored more fully. It might be expected that the global increase in terrorist acts might serve to increase this generalized fear.

To minimize and gain control of such fears, pre-departure training in stress inoculation therapy (SIT) is recommended. The expatriate would undergo a form of (SIT) [42] which would include exposure therapy in the form of systematic desensitization. Embracing the transactional model of stress and coping [22], SIT is based on the notion that exposing people to milder forms of stress, such that coping mechanisms will be bolstered as will the person’s confidence in using his or her coping repertoire [43]. SIT is intended to fortify an individual’s preparedness and develop a sense of mastery [43].

The use of virtual reality (VR) training offers a way to expose expatriates and their families to various aspects of the host country: culture, language, values, day-to-day living, etc. This technology can also be used as a type of exposure therapy for high-risk situations and terrorist threats. According to Hsu et al. [44], the use of VR offers many advantages:

Particularly during high impact, low probability events, appropriate personnel response relies upon the ability to perform their designated roles. Unforeseen psychological effects of stress brought about by unfamiliar environments or situations can impair decision-making and directly affect performance, leading to degradation of even routinely practiced skills. Disaster or public health emergency training scenarios incorporating real event elements (e.g., large crowds, damage to infrastructure, background noise, and visual and auditory cues) can better approximate real life conditions while retaining the advantages of a controlled environment. This increased practice realism enables responders to gauge their individual and/or team’s ability to execute tasks and decision-making under more closely representative conditions [44].

This could include exposure to unfamiliar social situations, reviewing security procedures, and identifying situations that activate coping mechanisms to enhance resilience. The use of virtual reality as a type of exposure therapy is being investigated in the treatment of victims of terrorism with PTSD [45,46]. This might also be incorporated into a pre-departure phase of proactive coping.

In many respects, pre-departure training in coping skills and strategies sets the framework for in-country and repatriation stages (Figure 1). The candidates have been assessed for their level of fear associated with deployment to a terror-endangered region and, if not excessively high, use proactive coping to build a resource base. Excessive, generalized fears may exclude a candidate from assignment.

### 5.2. In-Country Strategies

Some organizations, as a result of terrorism, and the risk of expatriates’ lives, are building security planning and procedures into their organizations [36]. This is critically important in this time of terrorist threats and attacks. However, individual expatriates need to be psychologically prepared as well. Aspinwall and Taylor [26] present a potentially useful framework for expatriate’s coping with living and working abroad, in a country under constant threat of terrorism. Proactive coping, or the efforts that a person would take in order to prepare for a perceived possible threat (such as terrorism), before it occurs, in an effort to prevent or modify it, if it should occur [27], can be taught to the expatriate before moving to the country (Figure 1). Thus, the expatriate has a coping repertoire ready for use in the host country should terror attacks occur. In addition, Hobfoll, Canetti-Nisim and Johnson [27] proposed that counseling pertaining to terrorism should occur that would focus on self-efficacy [31]. This counseling could be pro-active which could, possibly, protect against negative consequences after an attack or threat.

Expatriates on assignment must deal with stressors related to their work but also stressors emanating from the non-work domain. Bader, Reade and Froese [6] found that work stressors made the expatriate consider taking another position during the assignment, while non-work stressors soured the person on the country leading to the possibility of turnover. Other findings indicate that high levels of expatriate stress in terror endangered environments can have a negative impact on performance [47]. This is a point of intervention where coping and social support can make a difference. Support from coworkers and the organization are critical and depend to some extent on the expatriate’s level of sensitivity to terrorism [48].

We argue that effective coping strategies can help the expatriate deal more effectively with such cognitions relating to withdrawal behaviors, particularly turnover, and enhancing job performance. While it may not always be feasible to have psychologists available in country for stressful events, the expatriate should have psychological support and social support from the company to reinforce their coping efforts.

### 5.3. Repatriation Strategies

While there are studies that consider coping strategies during repatriation [35], no studies were identified that considered coping on returning from a high-risk, terror prone assignment. This is a significant gap in the literature since long-term health issues may not be immediately apparent upon return. We know that soldiers returning from war torn and terror prone environments often develop anxiety disorders and PTSD [49,50] (Figure 1). In many such cases, the symptoms do not emerge until long after returning, oftentimes for two years [51]. This is a cautionary tale for expatriates returning from terror-prone postings. At the very least, returning expatriates need a complete psychological assessment as soon as feasible after return and at appropriate intervals over the next two-year period. This poses challenges in light of the high turnover rate among repatriates [52].

Repatriates should be assessed for evidence of PTSD and other adjustment issues. Studies that focus on post-traumatic stress and coping [32,34] are suggestive although they do not focus specifically on repatriates. This research indicates that some sort of exposure therapy is beneficial for the amelioration of PTSD following a disaster or terrorist attack. Unlike PTSD treatment, expatriates would imagine the traumatic event. Then, they would proceed through graduated exposure, that is, gradual exposure to a fear hierarchy with the added procedures intended to produce physiological reactions that are incompatible with fear and anxiety. For example, when exposed to a traumatic event, an expatriate would perform muscle relaxation exercises that are incompatible with a stress reaction. Companies need to consider if in-house staff psychologists can handle these issues or if referrals are the preferred strategy for possible treatment interventions.

While many studies focus on career issues and knowledge transfer for repatriates [53], we argue that successful adjustment and psychological wellbeing are critically important. Coping strategies are an essential part of this adjustment experience. Repatriation involves unique stressors associated with the transition in the context of expectations that were anticipated before the expatriate left their home country: What is my position in the company? Is my support network still in place? How is my family coping with this adjustment? Is my time overseas recognized, valued, and appreciated? How has my compensation been adjusted? How has deployment in a terror-endangered location affected me? The uncertainties of repatriation can be stressful. It is also quite possible that the repatriate is not fully aware of the sources of their anxieties and adjustment issues.

## 6. Discussion

The rise of globalization and the increase in the occurrence of terror attacks has brought attention to the need to understand the behaviors and strategies that enable expatriates to live and work in countries that are under constant threat. These threats associated with working in a terror endangered setting represent a class of stressors that are qualitatively different from the usual adjustment issues associated with expatriate postings [6]. We present a heuristic model (Figure 1) of expatriate coping with terrorism that includes proactive coping and VR training in the pre-departure stage, problem and emotion-focused coping and social support for use in country, and desensitization procedures for coping with anxiety disorders and PTSD that might be manifest during and after repatriation. This appears to be the first study to examine coping strategies across all three stages of expatriation for candidates assigned to terror prone countries.

The use of proactive coping and VR represent unique contributions to pre-departure training for expatriates being sent on high-risk assignments. Proactive coping builds resources needed to deal psychologically with the fears and anxieties associated with living and working in terror prone regions. Such proactive coping efforts are intended offset or eliminate stressors before they become a problem [26]. VR training can be used so the prospective expatriate can become familiar with general milieu and also riskier aspects of the country environment. This is not intended to replace pre-departure country visits but can help the expatriate to visualize and deal with potential in-country scenarios.

There is a dearth of research on repatriation from terror prone regions among expatriates. This is a critical gap in the literature. We believe that such returnees will be prone to anxiety and PTSD issues long after they are back at work in their home country. We argue that psychological assessments should commence on return to the home country and then, again periodically over the next two years, since the disorders under consideration may take time to emerge. Exposure therapies represent a treatment modality that might be appropriate for returnees. Given the high turnover rate among repatriates [52], issues of company liability and health care continuity for debilitated returnees should be noted.

With respect to in-country experiences, some research looks at the coping behaviors of expatriate managers, in an attempt to understand the manager’s cross-cultural adjustment [23]. However, such studies did not focus on the effects of, or coping with, the constant threat of terrorism. Furthermore, the Hobfoll, Canetti-Nisim and Johnson [27] study investigated the impact of terrorism on those who are citizens of a country that is under constant threat of attack, although the participants in that study were not expatriates. Future research is needed that will focus on the impact of terrorism and the coping behaviors/strategies of those who are temporarily living and working in a foreign country and who are or might be risking their lives. Bader, Reade and Froese [6] produced the most germane research to date since it looks at withdrawal cognitions in relation to work and non-work domains among expatriates in terror-endangered countries.

Most of what we know about expatriate coping comes from interview data although some studies have used self-developed scales [54] derived from coping theory. This is not a major limitation but future studies should consider using reliable and valid coping measures to facilitate interpretation and generalization of results. Sample selection has also been an issue. Samples are typically composed of a small number of expatriates from numerous countries. This presents an issue since country of assignment has been identified as a significant variable in the coping literature [23]. We realize the logistical issues in identifying prospective participants and enlisting their participation. Asking expatriates in terror-endangered settings to participate in research studies adds another stressor to a potentially difficult environment.

We have focused primarily on the psychological coping mechanisms of individual expatriates. Many of these potential expatriates have spouses or significant others and possibly children as well. This raises concerns over family deployments in the face of terrorism. It might be expected that the presence of one’s family would be a support structure that would facilitate adjustment and enhance work performance. There is not much evidence to support the idea that the presence of family and children are associated with higher expatriate performance [47] in terror-prone countries. Nevertheless, the reality is that some families will be deployed to high-risk countries. Family members should be involved in the decision process and apprised of country risk scenarios. We recommend that they receive the same coping training as the expatriate.

We did not consider gender differences in coping with terrorism as an expatriate and no studies on this topic were identified. The closest study we found focused on Israel [54]. In a national probability sample, women reported significantly higher number of traumatic stress-related symptoms and PTSD symptoms in response to terrorist attacks [54]. Note that these finding relate to Israeli citizens and not expatriates. Koveshnikov et al. [55] reported an interaction effect between gender and the ability to appraise and express emotions: the influence of the latter on all three dimensions of cultural adjustment was somewhat stronger for male than female expatriates. In a study of Swedish managerial expatriates [56], however, men and women managers were more alike in coping than they were to Swedish citizens in general. Looking at gender in future research on coping in terror-endangered environments is encouraged.

## 7. Contributions and Implications

Our model draws attention to individual coping in terror-endangered countries based on the stage of expatriation (Figure 1). If terrorism is psychological warfare [57], we argue that psychological resources in the form of coping strategies need to play an important role in expatriate adjustment and performance. The extant literature is rather limited but there are many avenues for additional theoretical and empirical work. It would make sense for future empirical studies to examine each stage separately as a first step (cross-sectional approach). Subsequently, longitudinal research would be needed to test coping over the entire expatriation experience. Even small samples of expatriates studied over time would help us to understand the connection between and development of coping strategies across expatriation adjustment stages. We also need to know more about what coping strategies are successful in dealing with fear of terrorism as well as terrorist attacks and how this translates into expatriate performance and assignment completion.

The transitions between the stages of our model will be a challenge; for example, building coping resources before the assignment and then being confronted by the demands and stressors of being on-ground (e.g., terrorist threats). Ideally, this transition would be seamless but that seems unrealistic. Social and organizational support could make this transition more manageable. A combination of adjustment theory and conservation of resources [30] may help to explain such transitions as well. Expatriates should be aware of the possible loss of resources during transitions. In this way, some challenges can be anticipated and planned for. Successful coping when confronting unexpected events may bolster feelings of competency and enhance expatriate well-being.

Organizations and human resource departments have a critical role in recognizing the psychological factors that affect expatriate selection and adjustment as terrorism proliferates. Our work is intended to complement global safety and health models as well as the emerging literature on managing global human resources in hostile environments. All companies doing business on the global stage need terrorism preparedness plans [15]. Duty of Care [15,58] extends beyond providing for expatriates’ physical safety; psychological safety and well-being must also be recognized and given full consideration. We also acknowledge that, in addition to expatriates, our model is applicable to global business executives who travel a lot and may experience fears associated with terrorist threats and attacks.

Our proposed model can help organizations prepare expatriates to effectively deal with fear of terrorism and terrorist attacks. The ultimate goals center on selecting expatriates with the skills and temperament to advance company and personal goals in a secure and successful fashion. We believe that effective coping skills will contribute to assignment completion as well as expatriate well-being.

## 8. Conclusions

Coping with the fear of terrorism and dealing with terrorist attacks will be increasingly important in global human resource management. Proper psychological preparation, particularly coping skills of international assignees, will be necessary to ensure adjustment, performance, well-being, and assignment completion. Our approach indicates that different coping strategies are needed prior to the posting, during the assignment, and when returning home.
